# Essential oils prolonged the cut carnation longevity by limiting the xylem blockage and enhancing the physiological and biochemical levels

**DOI:** 10.1371/journal.pone.0281717

**Published:** 2023-03-07

**Authors:** Mayank A. Gururani, Amira K. Atteya, Abeer Elhakem, Abdel-Nasser A. El-Sheshtawy, Rasha S. El-Serafy

**Affiliations:** 1 Biology Department, College of Science, United Arab Emirates University, Al Ain, UAE; 2 Faculty of Agriculture, Horticulture Department, Damanhour University, Damanhour, Egypt; 3 Department of Biology, College of Sciences and Humanities, Prince Sattam Bin Abdulaziz University, Al-Kharj, Saudi Arabia; 4 Faculty of Agriculture, Environment and Bio-Agriculture Department, Al-Azhar University, Cairo, Egypt; 5 Horticulture Department, Faculty of Agriculture, Tanta University, Tanta, Egypt; Universidade Federal de Alfenas, BRAZIL

## Abstract

Postharvest characteristics, such as vase life and antimicrobial preservation of commercial cut flowers are some of the major determinants of their market value worldwide. Extending vase life while restricting microbial proliferation in cut flowers is an important challenge faced by floricultural researchers. This study evaluates the preservative efficiency of different essential oils used as additive solutions in prolonging the longevity of carnation cv. Madam Collette cut flowers and restricting microbial growth in them. Cut carnations were treated with four essential oils: geranium, thyme, marjoram, and anise at concentrations of 0, 25, 50, and 75 mg/L. While treatment with all the essential oils prolonged the longevity of the cut flowers, thyme and marjoram oils were most effective at concentrations of 50 mg/L each. The vase life of thyme-treated and marjoram-treated carnations almost doubled to 18.5 days and 18.25 days, respectively, as compared to untreated flowers. Treatment with essential oils also led to an increase in water uptake by the cut flowers enhancing their relative water content (RWC). It also restricted the sharp decline of chlorophyll and total carbohydrates content of the flowers during their vase life period. Morphological features of the stem bases of treated and untreated carnations were analyzed using scanning electron microscopy (SEM). The stem ends of geranium and anise-treated carnations showed less bacterial growth than untreated flowers and no apparent xylem blockage was observed even after nine days of treatment. Furthermore, the presence of essential oils also reduced lipid peroxidation and free radical generation as observed by malondialdehyde (MDA) and H_2_O_2_ quantification, respectively. It also led to increased production of total phenols leading to enhanced membrane stability. The use of thyme and marjoram essential oils as antimicrobial preservatives and green antioxidants appears to have promising applications in both the industrial and scientific sectors.

## 1. Introduction

Carnation *(Dianthus caryophyllus* L.) is one of the most popular cut flowers in the world, second only to rose in terms of commercial importance [1]. Several countries prefer carnations to roses and chrysanthemums for exportation due to their great keeping quality, broad range of forms and colors, and ability to resist long-distance transit. Carnations, roses, and chrysanthemums account for over half of the global cut flower trade. Prolonging the longevity of cut flowers is a major challenge for floricultural researchers. As a result, many studies pertaining to the enhancement of postharvest characteristics of cut flowers have been reported [2]. One of the major concerns affecting the longevity and quality of cut flowers is their water uptake and retention [3]. Due to limited water supply to the flowers and disruption of their water relationship, water stress reduces their vase life [3,4]. After harvesting, flowers undergo oxidative stress [5,6], leading to the generation of reactive oxygen species (ROS), membrane deterioration, and eventually cell death [7]. Alleviating oxidative injury is a determining factor in prolonging the vase life and ensuring the good quality of flowers.

The stem occlusion in cut flowers may occur due to microorganisms found in vase solution, physiological wound healing, and air embolism [4]. Lu et al., [3], Loubaud and van Doorn [4], He et al., [8] reported that the major reason of the xylem vessels blockage in cut flower stems is the bacterial growth, which in turn reduces the water conductivity, producing toxic metabolites and endogenous ethylene causing cell wall degradation [9]. In carnations, ethylene accelerates petal withering and petal browning and stops buds from opening where they are extremely sensitive to it. All Dianthuses must be maintained away from sources of ethylene during their postharvest period even if bud-stage flowers are less susceptible to ethylene than mature blooms are [2]. Thus, preventing microbial proliferation is a significant factor that helps in increasing the longevity of the cut flowers [10,11]. Generally, biocides and antimicrobial substances are used to control bacterial growth in vase solutions [12]. However, such substances are toxic and pose environmental and health hazard risks. Recent trends have shown increasing use of natural antimicrobial agents, which ensure an eco-friendly and inexpensive alternative in postharvest technology in the floral industry and sustainable agriculture.

Essential oils are aromatic liquids with a variety of biological and pharmacological properties, including antitumor [13], antidepressant [14], antioxidant, anti-inflammatory [15], insecticidal and/or repellent activity [16], antifungal [17], and antibacterial activity [18]. They are safe and eco-friendly alternatives for use as additives in vase solutions that improve the postharvest quality of cut flowers [19,20]. The antimicrobial properties of such oils can be attributed to various compounds present in them, such as sulfur-containing compounds in the aqueous phase [21] and oxygenated compounds, e.g., alcohols and phenolic terpenes. Phenolic compounds generally possess high antibacterial properties [22] and are the active compounds responsible for the preservative efficiency of essential oils. By virtue of these compounds, essential oils derived from geranium, thyme, marjoram, and anise are known to have antimicrobial properties. Geraniol and citronellol, both derived from the leaves and flowers of geranium, are monoterpenes with functional alcohol groups. Both compounds are known to possess microbial inhibitory and bactericidal activities [23]. Phenolic compounds, thymol, and carvacrol are the main ingredients of thymus oil and are also known to possess antibacterial and antifungal activity [24]. Anethole is the main active component in anise essential oil and has a broad range of antibacterial activity against both gram-negative and gram-positive bacteria [25]. Many existing studies have reported that essential oil supplementation to vase solution enhances factors such as water uptake by the cut flowers, relative fresh weight, and overall freshness of the cut flowers [26]. The essential oil derived from thyme plants has been reported to significantly improve the postharvest quality of gerbera [27,28], narcissus [29], and chrysanthemum cut flowers [30]. In another report, thymol was used to increase the vase life of Alstroemeria cut flowers [31]. Geranium oils have been reported to enhance the postharvest vase life of chrysanthemum [32,33], rose [34], and cloves [35] cut flowers. In another report, microbial contamination was significantly reduced upon vase solution supplementation with anise essential oils [36].

The current study aims to compare the efficiency of using geranium, thyme, marjoram, and anise essential oils in prolonging the vase life and the physiological and biochemical properties of carnation cut flowers.

## 2. Materials and methods

### 2.1. Flower collection and preparation

This experiment was performed at the vase life chamber, Horticulture Department laboratory, Agriculture Faculty, Tanta University, Egypt. The cut flowers of white carnation cv. Madam Collette (*Dianthus caryophyllus* L.) were brought from Floramax Farm company. The flowers were harvested at the fully open stage (the commercial stage of carnation flowers when the outer petals are fully expanded) [2] and trimmed to 40 cm in length. Their lower leaves were removed before recording their weight.

### 2.2. Plant cultivation

Geranium (*Pelargonium graveolens*), thyme (*Thymus vulgaris*), marjoram (*Origanum majorana*), and anise (*Pimpinella anisum*) plants were cultivated during the winter season of 2020 at Experimental Farm, Faculty of Agriculture, Tanta University, Tanta, Egypt located at 30° 47′ 18.00″ N, Lon 30° 59′ 54.61″ E latitude, sea level 8 m. The seedlings were thinned to one plant/hill. Geranium, thyme, and marjoram plants were harvested at the flowering stage and prepared for essential oil extraction, while anise plants were harvested on 1^st^ May 2021, and their fruits were cleaned and prepared for oil extraction.

### 2.3. Essential oils extraction

The essential oil of geranium, thyme, marjoram herb, and anise fruits were extracted by hydro-distillation for 3 hours using the Clevenger type apparatus. Anhydrous sodium sulfate was used to remove moisture from the essential oils. The oils were then stored in the dark at 4^˚^C for analysis and use.

### 2.4. GC-mass analysis

Oil composition was characterized using GC–MS analysis (Perkin Elmer; Model Clarus 580/ 560 S), having four capillary columns (30 m ×0.25 mm ID, film thickness 0.25 μm). Helium was used as the carrier gas at a flow rate of 1 mL/min and a three min solvent delay. Mass spectrometry was conducted in electron impact ionization mode at 70 eV, in scanning mode from 50 to 1000 m/z. The temperature program was as follows: source temperature was 200°C, MS injector transfer line temperature was 280°C, and the starting column temperature was 60°C for 1 min before ramping to 185°C, Cat 3°C/min and hold for 1 min, then Cat 9°C/min and hold for 2 min at 275°C. About 1 μL of oil sample was manually injected (at spilt ratio 1:20). The retention time and mass spectrum of essential oils were compared to those of standards. The NIST library was present in the GC–MS system, and information from existing literature was used to identify the active ingredients in the respective oils [37].

### 2.5. Preparation of preservative solutions and treatments

The stock solutions of the extracted oils were prepared as follows: 0.1 g of each crude oils were dissolved in 100 ml of Ethyl alcohol (99% v/v). Tween solution (1% v/v) was added while preparing the stock solutions to ensure complete dissolution of the oils. Working solutions of 0, 25, 50, and 75 mg/L of the oils were prepared from their respective stock solutions by appropriate dilution using distilled water. The treatments were performed in a complete randomized design, and each treatment had ten replicates (one flower/replicate). The procedure has been discussed in detail below. The flower quality and physiological traits were measured on days five and nine (when the longevity of untreated flowers was over).

### 2.6. Vase life

Cut carnation flowers were kept in jars containing 100 mL of the prepared preservative solution, supplemented with 5 g/L sucrose w/v, at 19 ± 2°C, 63 ± 5% relative humidity, and 12 h photoperiod at a light intensity of 10–12 μmol m^–2^ s^–1^ irradiance using white and cool fluorescent lamps. To prevent contamination and evaporation, the mouth of the bottle was covered using a plastic film [38]. The flowers were observed daily. Carnation longevity was estimated as the number of days required for 75% of the cut flowers to lose their turgor and ornamental value (characterized by wilting of the flowers).

### 2.7. Solution uptake and flower diameter

The solution uptake (in mL) was calculated as the difference between the volumes of solution at the beginning of the treatment and after cutting the wilted flowers. The diameter of cut flowers (cm) was measured at the full expanding stage of each treatment.

### 2.8. Relative Water Content (RWC)

Fresh leaves were collected and weighed. The weight of the fresh leaves was designated as W_fresh_. The leaves were then kept in distilled water at 4°C for 24 h. The weight of the saturated leaves was measured and designated as W_turgid_. Afterward, the leaves were oven-dried at 70°C for 48 h, and their weight was measured and designated as W_dry_. RWC was calculated according to the formula reported by Weatherley [39].


$$RWC\;\%=\frac{Wfresh-Wdry}{Wturgid-Wdry}\times 100.$$

### 2.9. Membrane Stability Index (MSI)

The second top leaves were used for the determination of MSI according to the protocol described by Sairam et al., [40] with modifications. Two leaf samples (0.1 g each) were collected and put in two 50 mL flasks, each containing 10 mL distilled water. The first leaf sample was kept at 40°C for 30 min, and its electric conductivity (C1) was measured using a conductivity meter. The second sample was kept in a water bath maintained at 100°C for 15 min. Its conductivity (C2) was measured accordingly. MSI was calculated using the formula of MSI = [1- (C1/C2)] X 100.

### 2.10. Total chlorophyll

Third leaves from the top of the stem were sampled to determine the chlorophyll content on day 9. The chlorophyll contents were measured by a chlorophyll meter (SPAD-502, Minolta Co., Japan) and were represented as SPAD values.

### 2.11. Total carbohydrates

The total soluble carbohydrates content of the leaves was measured on day 9. Leaf samples (0.1 g) were oven-dried at 60°C for 72 h and carbohydrate content was estimated according to the protocol reported by Herbert et al., [41].

### 2.12. Total phenols content

Total phenols (in terms of mg GAE g-1 dry weight) were estimated in the dried leaves spectrophotometrically (Model SM1200; Randolph, NJ, USA) by Folin–Ciocalteu’s reagent according to the method reported by McDonald et al. [42]. Gallic acid was used as the standard.

### 2.13. Malondialdehyde Determination (MDA)

Analysis of MDA concentration was used to measure the extent of lipid peroxidation, according to the method described by Heath and Packer [43], with some alterations. About 0.5 g sample of fresh leaves was combined with 5.0 mL of trichloroacetic acid (TCA) (5% w/v) and centrifuged at 12,000 g for 10 min at 4°C. A total of 2 mL of the extract was mixed with 2 mL of thiobarbituric acid (TBA) (0.6%) and heated in a water bath (95°C) for 10 min. Absorbance was measured at wavelengths of 532 and 600 nm. The following formula was used for MDA calculation: MDA content (mmol kg^–1^) = 6.45 × (A532 − A600) − 0.56 × A450.

### 2.14. Hydrogen Peroxide Measurement (H_2_O_2_)

The protocol reported by Patterson et al. [44] was used to quantify H_2_O_2_ levels in carnation leaves. Leaf samples (0.5 g) were pulverized in 6 mL of cooled acetone (100% v/v) and centrifuged at 12,000 g for 10 min at 4°C. About 1 mL of the supernatant was mixed with 0.1 mL of Ti(SO_4_)_2_ solution (5% w/v) and 0.2 mL of concentrated NH_4_OH solution, respectively. The mixture was then centrifuged at 3000 g for 10 min. dissolved in 4 mL of H_2_SO_4_ (2 M). The optical density of the solution was measured spectrophotometrically at 412 nm.

### 2.15. Visualization of Stem Bases Using Scanning Electron Microscopy (SEM)

The xylem at the base of cut stems was examined using scanning electron microscopy (TESLA BS- 300). Stem samples were collected on day 9. Cross-sectional segments of the stem bases were obtained using razor blades. Segments were fixed in FAA solution (90 mL: 5 mL: 5 mL of formalin (37–40%), alcohol (70%), and acetic acid) according to the protocol reported by Li et al. [45] after which they were dried at the critical point of CO_2_ (Balzers CPD-020) and coated with gold (30 nm) in a sputter coater (Balzers SCD-040). The segments were then examined and photographed.

### 2.16. Statistical analysis

The data was analyzed using a randomized complete design with ten replicates per treatment. The average of the treatments was compared for significance by ANOVA analysis according to Gomez and Gomez, [46] using COSTAT program. The comparison within means was evaluated by the Duncan test at *P ≥* 0.05 and results were presented as mean values ±SE.

## 3. Results

### 3.1. Essential oils composition

**Fig 1:**
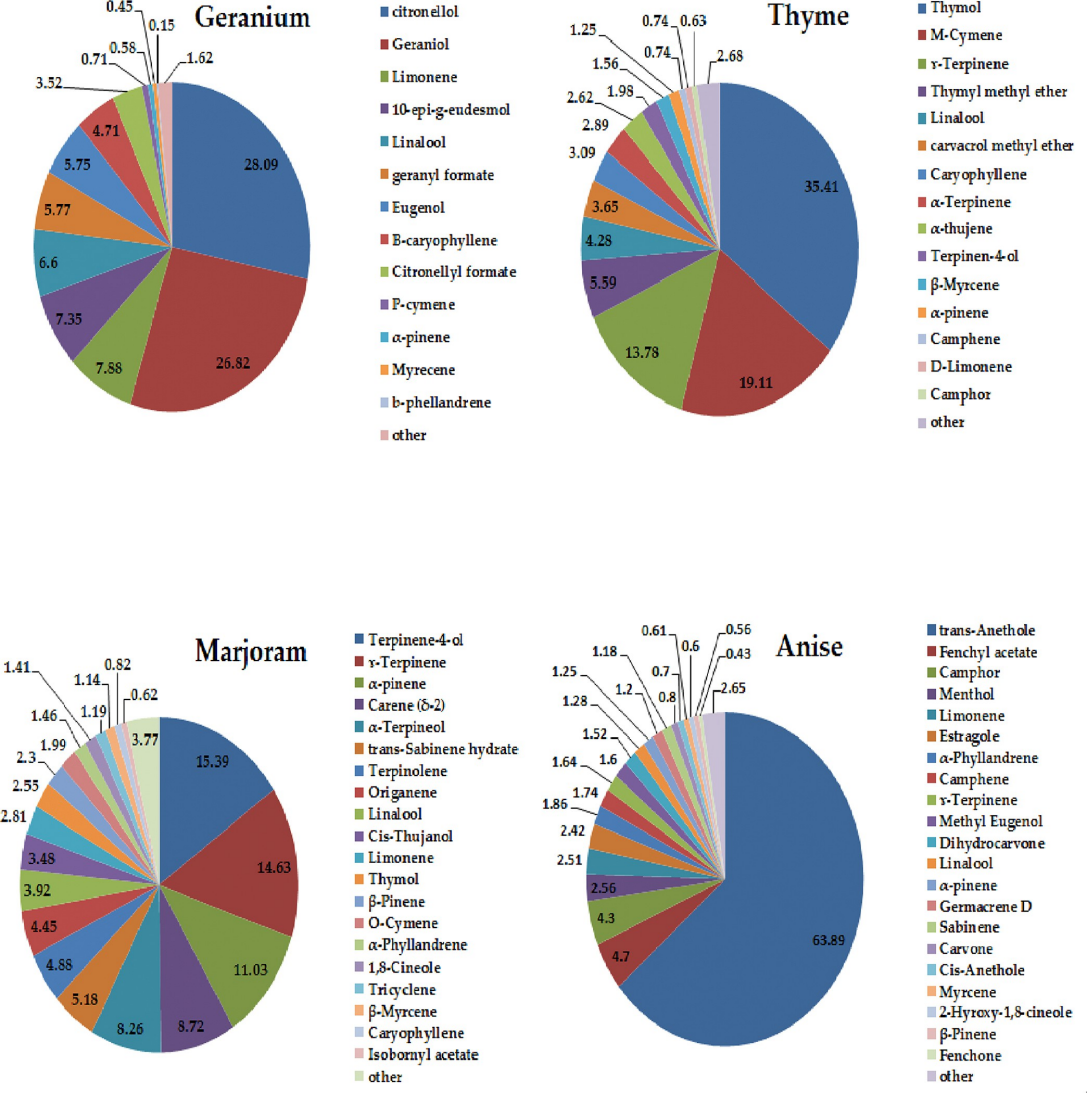
GC-mass analysis of geranium, thyme, marjoram, and anise essential oils.

The chemical composition of geranium, thyme, marjoram, and anise essential oils as identified by GC-MS analysis is presented in Fig 1. The main components of geranium oil are citronellol (28.09%), geraniol (25.82%), limonene (6.88%), 10-epi-g-eudesmol (6.35%), linalool (5.60%), geranyl formate (4.77%), eugenol (4.75%), b-caryophyllene (3.71%) and citronellyl formate (3.52%). Together these compounds formed about 89.49% of the geranium oil. Likewise, the main active components of thyme essential oil were identified as thymol (35.41%), m-cymene (18.11%), ɤ-terpinene (12.78%), thymyl methyl ether (5.59%), carvacrol methyl ether (3.65%); together forming 88.32% of the total oil. The chemical composition of marjoram essential oil was found to be terpinene-4-ol (21.39%), γ^–^terpinene (14.63%), α^–^pinene (11.03%), α^–^terpineol (10.26%). carene (δ-2) (8.72%), trans-sabinene hydrate (5.18%). GC-mass analysis showed that the major components of anise essential oil, were trans-anethole (63.89%), fenchyl acetate (4.70%), camphor (4.30%), menthol (2.56%), limonene (2.51%) and estragole (2.42%).

### 3.2. Vase life and solution absorption

**Fig 2:**
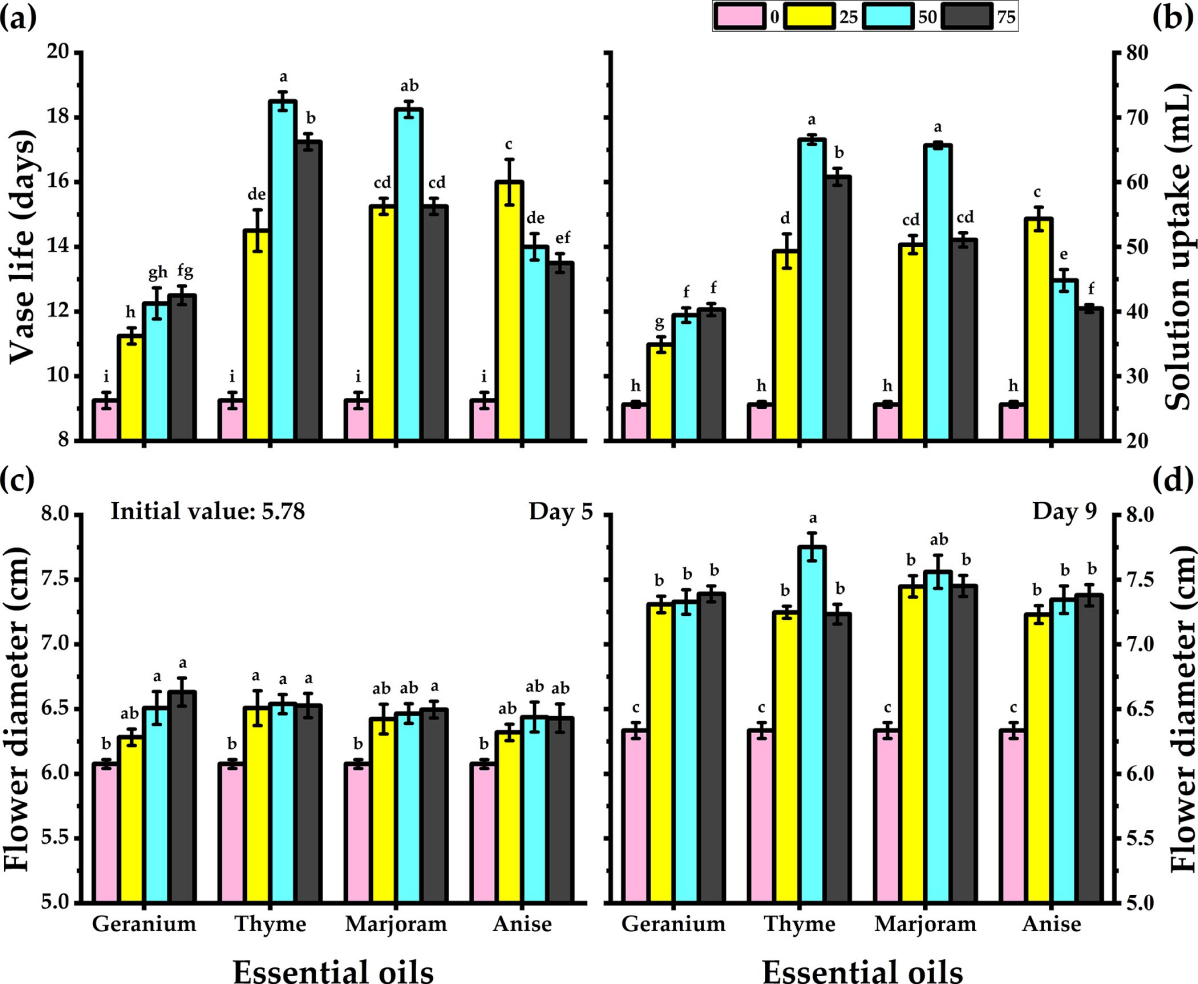
Response of carnation cut flowers to geranium, thyme, marjoram, and anise essential oils at 0, 25, 50, and 75 mg/L concentrations as preservative solutions on the vase life (a), solution uptake (b), and flower diameter (c and d) at day 5 and day 9 of vase life period. Bars with the same letters are not significantly differed at *p* ≤ 0.05 level.

Treatment with the essential oils effectively extended the longevity and increased the solution uptake of the cut carnations cv. Madam Collette flowers as compared to the untreated ones (Fig 2A and 2B). Flowers showed the longest vase life and maximum absorption of the preservative solution when treated with thyme oil, followed by marjoram oil, anise oil and geranium oil. The optimum concentration of the essential oils was found to be 50 mg/L. An addition of 50 mg/L thyme oil in the preservative solution resulted in a 100% increase in the vase life of the cut flowers i.e, it increased from 9.25 days in untreated flowers to 18.50 days in treated flowers. Solution uptake also increased from 25.65 mL in untreated flowers to 66.58 mL in thyme oil-treated ones. Any further increase in concentration of the oils led to slight decrease in the longevity of the flowers, albeit still more effective than untreated flowers.

### 3.3. Flower diameter

The cut carnations treated with thyme preservative solution exhibited more petal expansion. While untreated flowers exhibited an increase of 11.20% in petal expansion from day 5 to 9, thyme oil-treated flowers showed an increase of 13.10% (Figs 1D and 2C). A marked increase in flower diameters was observed at all concentrations of essential oils. An increase in petal expansion and flower diameter in thyme-treated flowers continued well up to 9 days. Consequently, their diameter was measured to be 7.75 cm. However, the highest petal expansion in untreated flowers was observed on day 5 and the maximum flower diameter was 6.08 cm. Among all the essential oils, anise oil was found to be the least effective. At 25 mg/L, it led to the formation of flowers with a 7.23 cm diameter, albeit higher than the diameter of untreated carnations.

### 3.4. Relative water content

**Fig 3:**
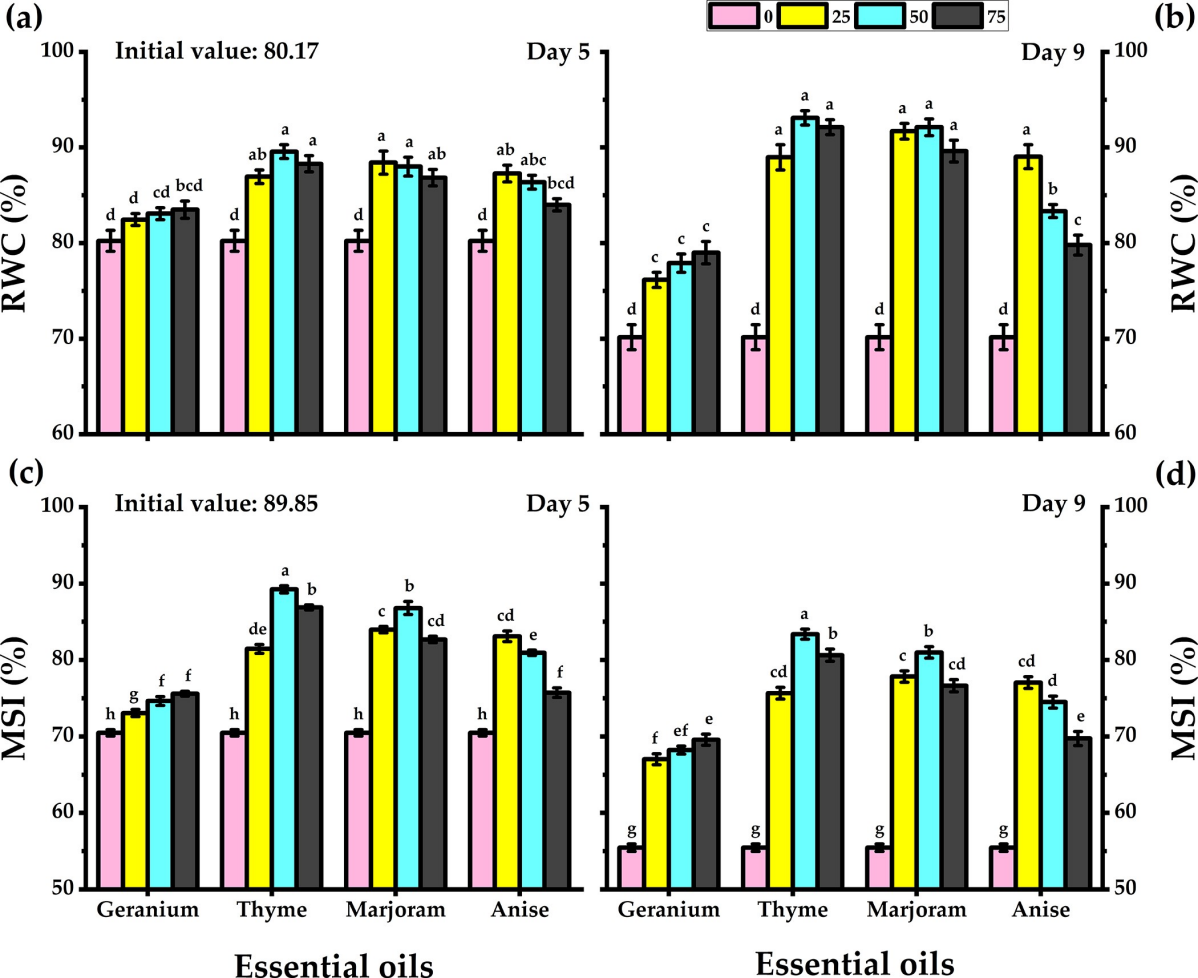
Response of carnation cut flowers to geranium, thyme, marjoram, and anise essential oils at 0, 25, 50, and 75 mg/L concentrations as preservative solutions on the RWC (a, b), and MSI (c, d) at day 5 and day 9 of vase life period. Bars with the same letters are not significantly differed at *p* ≤ 0.05 level.

The leaves of cut carnations treated with all types of essential oils presented significantly higher RWC levels than those of untreated carnations on both day 5 and day 9. The highest RWC values were obtained by thyme and marjoram oil treatments (Fig 3A and 3B). Treatment with geranium oil had the least effect on RWC levels. Though all concentrations of the oils led to higher RWC, 25 mg/L of thyme oil seemed to be optimum. Throughout their vase lives, while the RWC of untreated carnations decreased gradually, it remained constant in those treated with essential oils.

### 3.5. Membrane stability index

The presence of essential oils in vase solutions maintained the membrane integrity of carnation leaves which was evaluated in terms of membrane stability index (MSI). MSI values of treated and untreated flowers are shown in Fig 3C and 3D. The highest MSI value was observed with 50 mg/L of thyme oil. The level of 50 mg/L significantly produced the highest MSI values than other levels on both day 5 and day 9. Carnation flowers preserved in 50 mg/L thyme solution showed the lowest reduction in MSI value; only a 7% decrease in membrane integrity was observed. Whereas MSI values show that membrane integrity was reduced by 27% from day 5 to day 9 in untreated flowers.

### 3.6. Total chlorophyll

**Fig 4:**
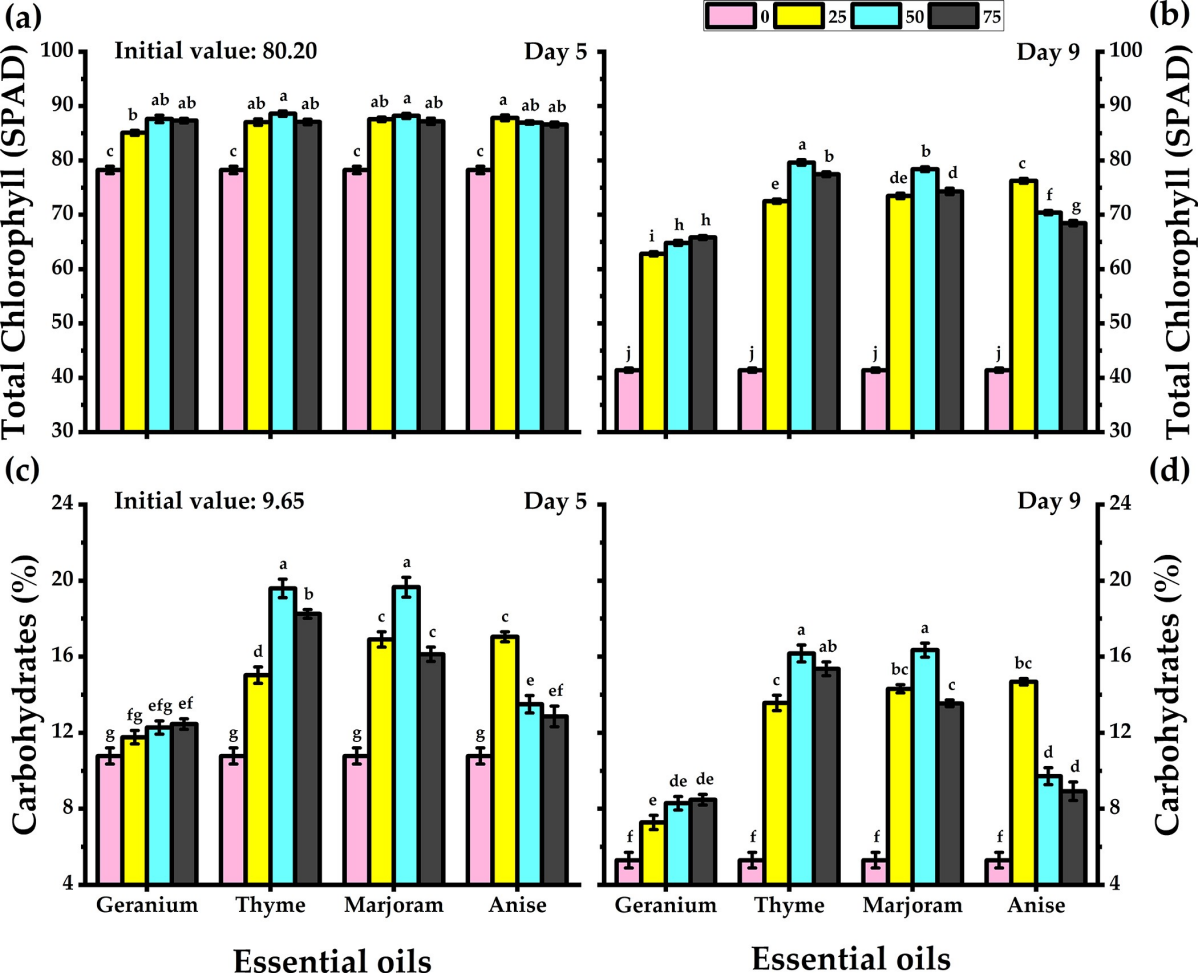
Response of carnation cut flowers to geranium, thyme, marjoram, and anise essential oils at 0, 25, 50, and 75 mg/L concentrations as preservative solutions on the total chlorophyll (a, b), and carbohydrates content (c, d) at day 5 and day 9 of vase life period. Bars with the same letters are not significantly differed at *p* ≤ 0.05 level.

Essential oil-treated carnations showed significantly higher chlorophyll content on both days 5 and 9 of their vase life. The highest chlorophyll content was observed in thyme oil-treated carnations, whereas geranium oil-treated carnations had the lowest chlorophyll content (Fig 4A and 4B). All oil concentrations-maintained chlorophyll content in cut flower leaves, and 50 mg/L was the most ideal. Thyme oil treatment (50 mg/L) produced the highest chlorophyll content (79.63 SPAD) and restricted chlorophyll degradation during the vase life period. On the other hand, untreated flowers presented the lowest chlorophyll content (78.3 and 41.4 SPAD on day 5 and 9, respectively).

### 3.7. Total carbohydrates

Carbohydrate content was observed to be higher in essential oil-treated flowers (Fig 4C and 4D). Thyme oil-treated flowers had 13.9% and 14.5% carbohydrates on day 5 and 9, respectively. Whereas geranium oil-treated flowers had 11.8 and 7.3% on day 5 and 9, respectively. While 50 mg/L of all essential oils led to higher carbohydrate content, thyme oil was the most effective in this regard. The lowest carbohydrates level was noticed by untreated flowers which exhibited 10.78 and 5.3% for day 5 and 9, respectively.

### 3.8. Total phenols content

**Fig 5:**
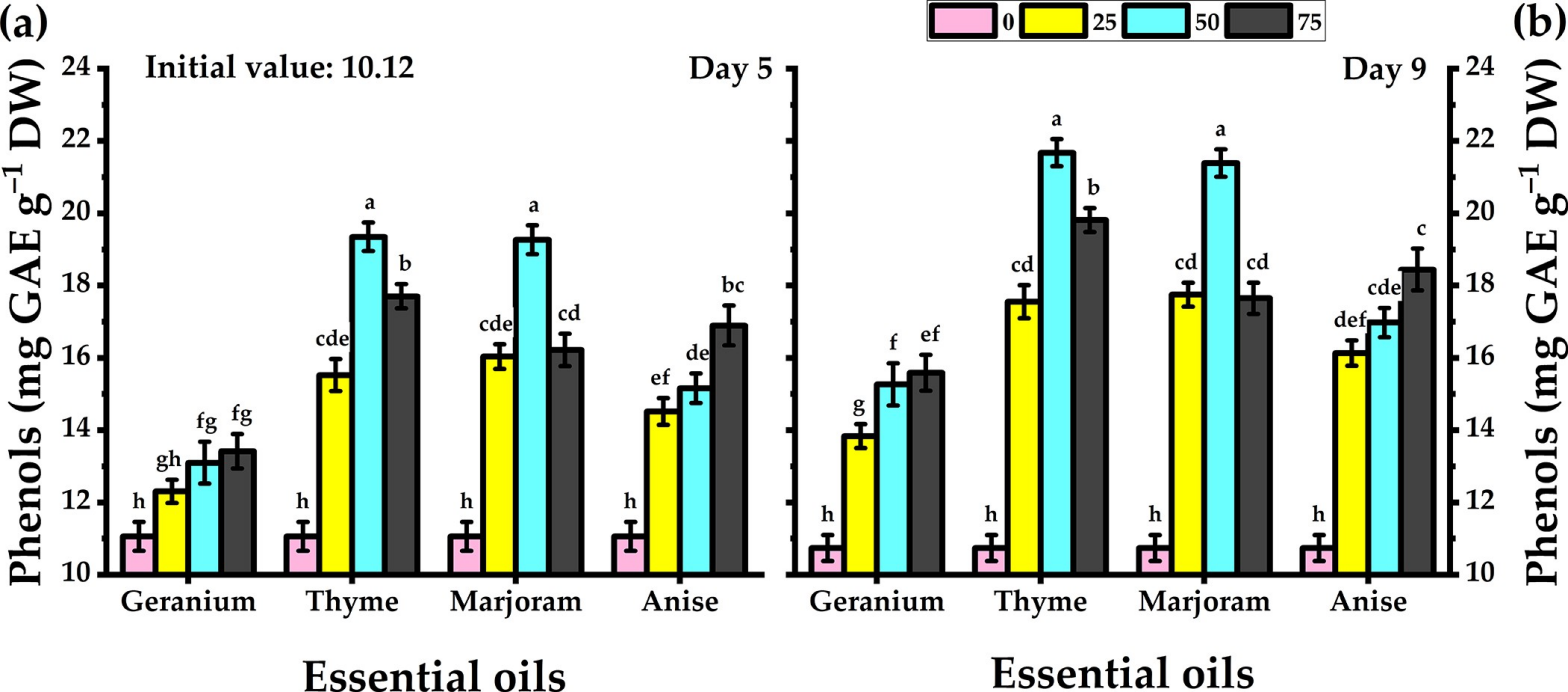
Response of carnation cut flowers to geranium, thyme, marjoram, and anise essential oils at 0, 25, 50, and 75 mg/L concentrations as preservative solutions on phenols content at day 5 and day 9 of vase life period. Bars with the same letters are not significantly differed at *p* ≤ 0.05 level.

During the vase life evaluation period, total phenol content in carnation leaves was observed to increase following treatment with essential oils, whereas, in untreated carnations, it had reduced by day 9. (Fig 5A and 5B). While treatment with all essential oils increased the phenol content at day 9, the highest levels were observed upon treatment with 50 mg/L thyme (19.35 and 21.68 mg g^-1^ GAE for days 5 and 9 respectively).

### 3.9. MDA and H_2_O_2_ content

**Fig 6:**
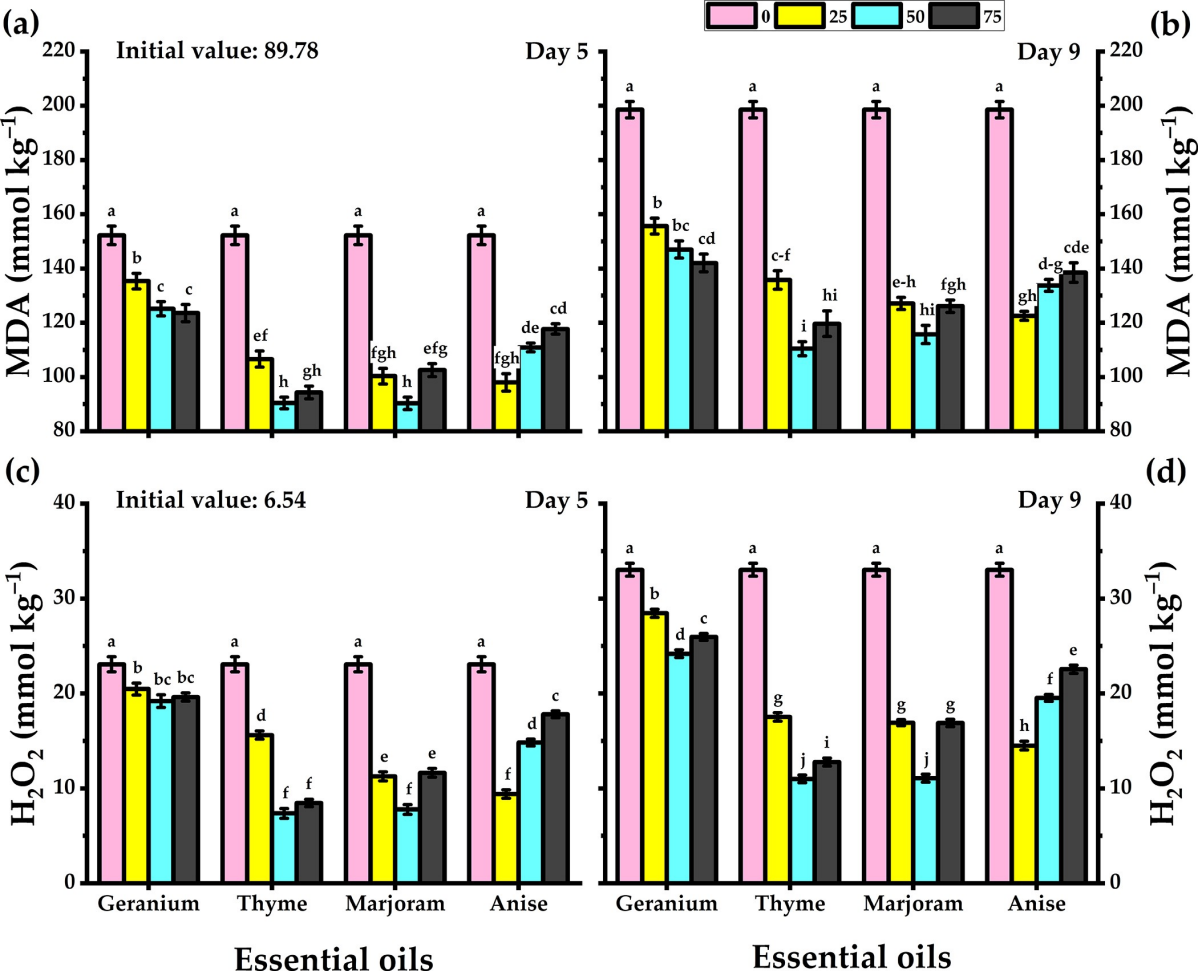
Response of carnation cut flowers to geranium, thyme, marjoram, and anise essential oils at 0, 25, 50, and 75 mg/L concentrations as preservative solutions on the MDA (a,b), and H_2_O_2_ at day 5 and day 9 of vase life period. Bars with the same letters are not significantly differed at *p* ≤ 0.05 level.

Treatment with essential oils led to a reduction in MDA and H_2_O_2_ levels (Fig 6). Significant reduction was observed with thyme and marjoram treatment on both days 5 and 9. Treatment with thyme and marjoram oils at their respective concentrations of 50 mg/L was most effective in reducing MDA and H_2_O_2_ concentrations in the flowers.

### 3.10. Scanning Electron Microscopy (SEM)

**Fig 7:**
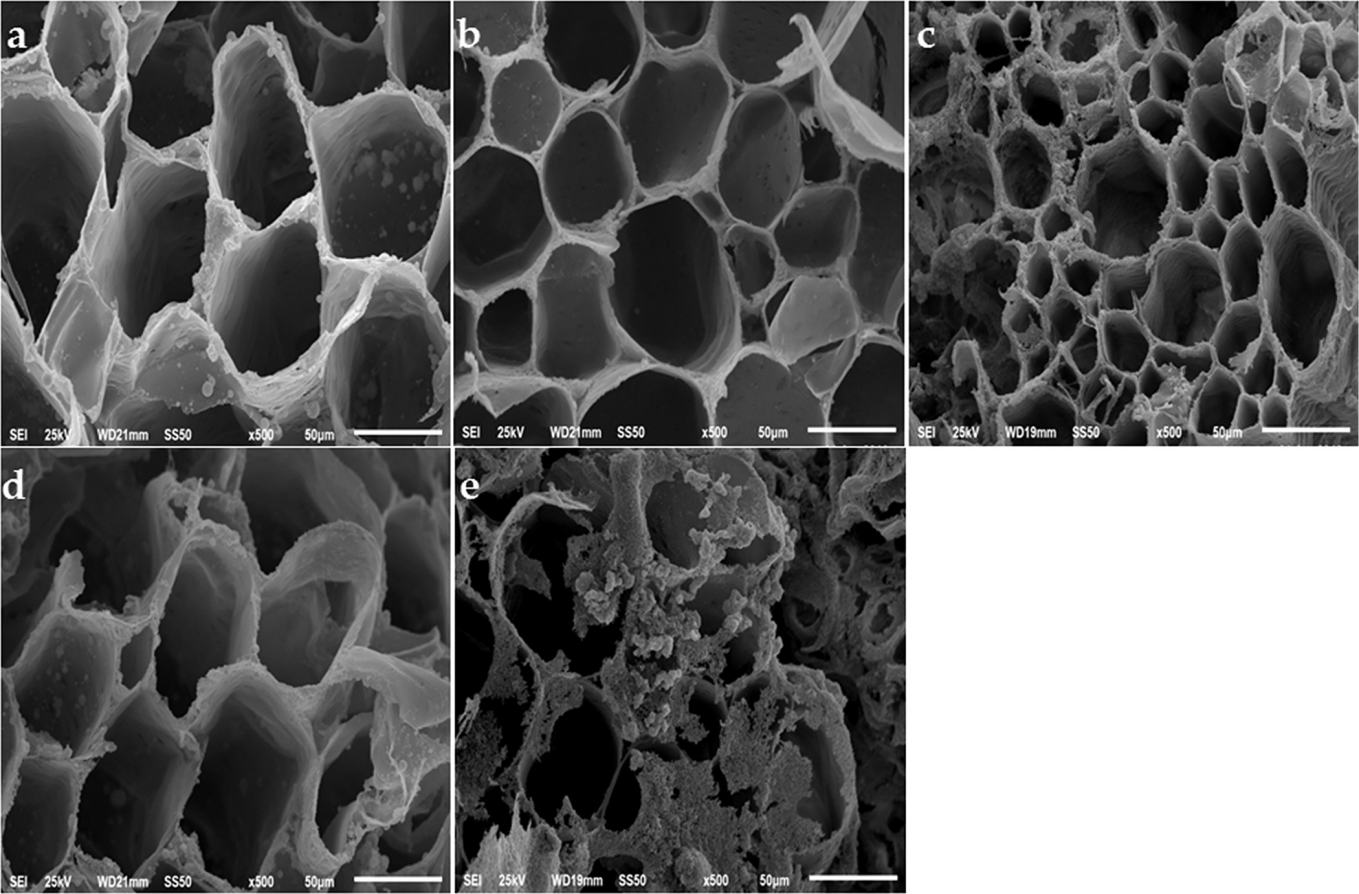
Carnation stem cross section at the base of cut flower stems a: geranium, b: thyme, c: marjoram; d: anise, and e: untreated flowers. The section was made at the end of the vase life of each treatment (Magnification:1500x).

The images obtained from scanning electron microscopy showed the morphological features of xylem cells in the stem bases of essential oil-treated and untreated flowers (Fig 7). While untreated flowers had blockages and bacterial buildup in their xylem vessels, treated flowers had much clearer vessels and significantly lower bacterial presence (unpublished data). Thyme and marjoram oils were most effective in preventing microbial growth (unpublished data). Geranium and anise essential oil could only partially reduce the growth of bacteria and xylem blockage in the stem end.

## 4. Discussion

Microbiological invasion in the preservative solution is one of the major factors determining the vase life of cut flowers and reducing water relations. Internal ethylene and toxic compounds are produced by microorganisms, which also cause stem blockage and hastened flower petal aging. Hydraulic conductivity in cut flower stems is minimized when microorganisms proliferate in the vase solution [36]. Using essential oils as antimicrobial agents in preservative solutions is effective in minimizing bacterial growth and development while extending vase life [47,48]. Maintaining the water relationship during the postharvest stage has been identified as a critical factor in extending the longevity of cut flowers, whereas a disturbed water balance causes their senescence.

Essential oils used in this study extended the vase life of cut flowers, improved the water uptake of cut carnations while maintained an elevated RWC compared to untreated flowers. An increase in RWC following essential oil treatments has been reported by Ezhilmathi et al., [5]; Solgi et al. [12]; Solgi and Ghorbanpour, [49]. The longevity and quality of cut flowers have been enhanced upon treatment with herbal essential oils [30,33,49,50]. The water uptake of thyme-treated flowers (50 mg/L) was 40 mL more than those of untreated flowers. More the water uptake, the higher the RWC which in turn aids in carbohydrate metabolism in the cells of the petals providing the required energy for sustenance and respiration [51–54]. Flower diameter is considered while determining the quality and market value of cut flowers. In the current study, carnation flowers treated with essential oils presented more petal reflection with higher flower diameters than untreated flowers (Fig 2c and 2d).

Essential oils derived from dill, artemisia, and thyme were reported to increase the petal reflection and flower diameter of cut chrysanthemums [33,50]. Additionally, thyme and marjoram essential oils treatments prevented a sharp decline in the chlorophyll content of the cut carnations. Similar findings were reported with chrysanthemum flowers where treatment with essential oils ensured higher chlorophyll content [33,50]. Essential oils are rich in antioxidants which prevent the degradation of chlorophyll [31]. This in turn can be attributed to an increase in glucose synthesis and cellular activity [55]. Chlorophyll degradation is minimized when the glucose content is increased via osmotic pressure and respiration management [56–58]. Essential oils have antimicrobial property which prevents the growth of microbes that possess chlorophyllase activity, thereby preventing the degradation of chlorophyll [59,60]. In the current, the increase in chlorophyll content is followed by an increase in total carbohydrates in carnation leaves. Such results were obtained by Lise et al., [61]; Atteya, et al., [62]; Rong-Hua et al., [63]. Throughout the vase life period, respiration is the main physiological function, which results in a decrease in carbohydrates accumulated in the cut flower leading to wilting [33].

Water stress and vascular blockage increased free radicals in chloroplasts [50]. As a result of the antioxidant property of the essential oils, [64] revealed that essential oils preserved or enhanced the chlorophyll in Lisianthus cut flowers. The free oxygen radicals (such as H_2_O_2_) are responsible for lipid peroxidation which causes heightened MDA production [50,65]. The H_2_O_2_ and MDA accumulation is an indicator of cellular membrane degradation. Treatment with essential oils significantly minimized H_2_O_2_ levels in Madam Collette carnations leaves as compared with untreated carnations, and 50 mg/L thyme and marjoram oils outperformed the others in this respect. High MDA content in plant tissues is considered as a signal of physiological tolerance and senescence [66]. In this study, thyme and marjoram oils were found to be more effective in reducing MDA content than geranium and anise. Essential oils extracted from plant herbs are rich in phenolic ingredients that have the capability of decreasing membrane lipid oxidation, MDA level, and reactive oxygen species removal [67]. High MSI and low MDA levels indicated the long vase life of cut chrysanthemum [60]. Essential oils significantly enhance total phenol content in carnation leaves as compared to untreated plants. One of the major factors that caused early petals aging is the free oxygen species released from hydrogen peroxide decomposition, phenols are antioxidants that neutralize the harmful oxygen release of hydrogen peroxide, and their activity slows the aging of petals [68,69].

Flowers suffer from wilting when they fail to absorb water, which can be caused by the proliferation of bacteria in the cambial tissues of the stem [8]. Microbial growth and accumulation of microbial debris cause blockages in the xylem vessels of the floral stems, which disrupts water conductivity resulting in wilting, petals drying, and early aging [70]. Essential oils have antifungal and antibacterial properties, which help to extend the vase life of cut flowers [71]. Further antimicrobial substances decrease the cellular damage caused by free radicals and accordingly improve the longevity of cut flowers [35]. The antimicrobial properties can be attributed to the presence of active ingredients and alcohol bases which are found at low concentrations in essential oils [72–74]. The chemical composition, synergistic interaction, and functional groups existing in the active ingredients are the main factors that determine the activity of the essential oil [75]. Terpene is one of the active compounds in essential oils that have the ability to restrict oxidative stress caused by an accumulation of reactive oxygen species [76]. Terpenoids are divided into aldehyde (citronellal and citral), alcohol (geraniol, linalool, terpineol, borneol, carveol, menthol bisabolol, and citronellol), ether (eucalyptol), ketone (carvone and camphor), hydrocarbon (pinene, phellandrene, and limonene), and phenol (thymol and carvacrol), groups. The best antimicrobial activity is shown by the compounds identified with polar functional groups and low molecular weights. The presence of these compounds can enhance antimicrobial activity as they can easily penetrate the outer bacterial membranes. Eugenol is a low molecular weight phenolic compound which high antibacterial activity [77]. Phenolic compounds (e.g. the oxygenated terpenes “terpenoids”) have preferable antimicrobial activity as compared with hydrocarbons [77]. Hydroxyl groups present in thymol, eugenol, terpineol, and carvacrol, are extremely reactive and establish hydrogen bonds with target enzyme active sites, rendering them inactive [78,79], and leading to cell membrane malfunction or rupture.

Thymol, cymene, and carvacrol are the main constituents of thyme essential oil and have strong antibacterial and antifungal effects [24]. Terpinene-4-ol, γ-terpinene, and α-terpineol are the main constituents of marjoram essential oil [80]. Geraniol, citronellal, and linalool are the main active ingredients in geranium essential oil, while the main active compound in anise essential oil is anethole. The antimicrobial activity of geraniol, citronellol, and geranyl acetate are shown to be correlated with their capacity to hurt the membrane integrity [81], proteins denaturation, and interfere with cell lysis and metabolism [82]. The antioxidant activity of marjoram essential oil can be attributed to a high concentration of terpinene-4-ol, γ -terpinene, and γ -terpineol, which is also considered to be strong scavengers of free radicals [83]. Solgi et al. [12] reported that thyme oil application is efficient in extending the shelf-life of cut gerbera due to its antibacterial property, while Habibi et al. [84] reported that marjoram essential oil exhibits high antibacterial activity which may be due to the linalool compound. Cymene, which constitutes 18.11%, is an active compound in thyme essential oil (Fig 1). It inhibits oxidative stress by elevating the activity of the antioxidant enzymes [85]. A minimal bacterial count was detected following cymene application, but more antimicrobial activity was detected when cymene was accompanied by 4-terpineol, linalool, α-terpineol which may be due to their synergistic effects.

In the current study, carnation cut flowers treated with all concentrations of anise oil also showed prolonged vase life as compared to untreated flowers. Anethole in anise oil is known to possess antibacterial activity against both gram-positive and gram-negative bacteria [25]. Carvacrol is known to be effective against a wide range of bacteria as well [86]. Carvacrol modifies the fatty acids structure of bacteria cell membranes causing fluidity and permeability, and exhausts the ATP of bacterial cells [87,88]. Methyl carvacrol, citronellol, thymol, and menthol compounds cause the cell membrane expansion, allowing for passive ion transport between the enlarged phospholipids [88,89], or represses the secretion of toxins [90]. Carvacrol can also inhibit the synthesis of the protein Flagellin, which is required for the motility of bacteria [88]. Eugenol elucidates its antimicrobial activity by modifying the outlying fatty acid in the bacterial cell membrane. Furthermore, it has the ability to destroy histidine, amylase, ATPase, proteases, and carboxylase bacterial enzymes [91]. Citronellal has been demonstrated to alter hydrophobicity and damage membrane integrity, allowing K^+^ ions to flow out [23]. The active ingredients in the essential oils are capable of penetrating the cell wall and cytoplasmic membranes of fungi and disrupting them [92].

## 5. Conclusions

Essential oils derived from geranium, thyme, marjoram, and anise have the ability to maintain the quality and prolong the vase life of cut carnations cv. Madam Collette. Thyme and marjoram oils were most effective followed by geranium and anise oils. The essential oils, by virtue of their active constituents, were capable of inhibiting microbial growth in the vase solution and stem ends of the cut flowers. They helped in maintaining water balance and increasing water uptake, improving antioxidant levels while reducing lipid peroxidation and H_2_O_2_ generation, and preserving the cellular membrane integrity (MSI). The optimum preservative concentration of essential oils was found to be 50 mg/L. These essential oils are eco-friendly alternatives to many toxic preservatives currently used in the cut flower market. Based on this study, we recommend thyme and marjoram oils at 50 mg/L for use as a preservative solution for commercial application in cut carnations.

## Supporting information

S1 File (XLSX)
